# Beyond the Corporatization of Death Systems: Towards Green Death Practices

**DOI:** 10.1177/10541373211006882

**Published:** 2021-04-07

**Authors:** Mark Shelvock, Elizabeth Anne Kinsella, Darcy Harris

**Affiliations:** 1 Western University, London, Ontario, Canada; 2 Faculty of Medicine and Health Sciences, McGill University, Montreal, Quebec, Canada; 3 King’s University College, Western University, London, Ontario, Canada

**Keywords:** green death practices, death systems, funeral industry, ecological crisis, death care

## Abstract

One less explored area of research concerns the response to the ecological crisis through environmentally sustainable death practices, which we broadly define in this paper as ‘green death practices’. In this paper, interdisciplinary research and scholarship are utilized to critically analyze death practices, and to demonstrate how contemporary Westernized death practices such as embalming, traditional burial, and cremation can have harmful environmental and public health implications. This paper also investigates the multi-billion-dollar funeral industry, and how death systems which place economic growth over human wellbeing can be socially exploitative, oppressive, and marginalizing towards recently bereaved persons and the environment. Death-care as corporatized care is explicitly questioned, and the paper provides a new social vision for death systems in industrialized Western societies. Ultimately, the paper advocates for how green death practices may offer new pathways for honoring our relationships to the planet, other human beings, and even our own deepest values.

While scholars from across the sciences and humanities are grappling with the impacts and challenges of the ongoing climate breakdown, one less explored area concerns the response to the ecological crisis through environmentally sustainable death practices, which we broadly define in this paper as ‘green death practices.’ Green death practices have also been referred to as “woodland, natural, or ecological burials” (Brennan, 2014, p. 233). We have adopted the term ‘green’ to explicitly connect these death practices to ongoing political discourses around social progressivism, environmental sustainability, non-violence, social justice, and ecological conservation. Additionally, the term ‘practices’ is used to illustrate how environmentally friendly choices around funerals can extend beyond burials, and incorporate countless actions, rituals, and ceremonies that are embedded within broader socio-cultural contexts. This topic is of importance because green death practices offer the potential to create better death systems to support our loved ones and ourselves when we encounter the inevitable. This is eloquently captured by Butler (2004), who states “loss has made a tenuous ‘we’ of us all” (p. 20).

When researching topics within the field of thanatology, it is fruitful to utilize an interdisciplinary approach, as interdisciplinary perspectives help us integrate various conceptualizations for multidimensional experiences around dying, death, funerals, bereavement, loss, grief, and other thanatological phenomena (Morgan & Morgan, 1988). As such, this paper weaves research and scholarship from across the natural sciences, social sciences, and humanities to critically analyze death practices. This analysis adopted a critical theoretical lens which emphasizes the social values and contexts of death practices, and which was productive in making visible green death practices as an important socio-cultural issue within Western society. An interdisciplinary approach was productive in illustrating the complexity of knowledge surrounding death practices, elucidating a range of vantage points, and for outlining advantages and disadvantages associated with current mainstream death practices versus green death practices.

We begin by illustrating important individual and socio-cultural considerations around death practices to demonstrate their historically, culturally, and socially embedded nature. Subsequently, we explicitly question the contemporary multi-billion-dollar death industry that controls death practices within Western societies, as this death system can result in social oppression of the bereaved during what is arguably one of the most vulnerable times of a person’s life. The categorization of ‘Western society’ is used to characterize the socio-political structure of capitalism, which is constructed upon industrialization, market-driven economies, and accompanying philosophical values which are not limited to any geographic place or country (Harris, 2010). Additionally, the paper outlines how capitalism is fueling the current ecological crisis, and critically analyzes toxic traditions of death practices within Western society that are contributing to individual, communal, and collective ecological harms. This article investigates ecologically conscious alternatives for death practices and argues that green death practices can serve as a new horizon of possibility in creating a more livable and equitable world. The paper ultimately attempts to deepen our individual and collective reflections.

## Death Practices: Individual and Socio-Cultural Considerations

While this research focuses around death practices that are directly related to the loss of a loved one, it is essential to identify that loss is ultimately whatever a person says it is, and the death of a loved one can result in numerous types of other losses. Loss is any experience where it is impossible to return to life as it once was, as there has been a change in perception, circumstance, or an experience surrounding a shattering life event (Harris, 2020a). Bereavement can be defined as the state in which people experience the loss and/or deprivation of a subjectively valued person, relationship, object, or thing (Corr et al., 2019). Grief is the instinctive response to bereavement and loss (Harris, 2020b; Harris & Winokuer 2016), and the grieving response affects a bereaved person in all dimensions of their existence simultaneously (Attig, 2011). Grief is often a life-long process that is unbounded by a specific amount of time (Harris, 2010; Shelvock, 2018), and while death may end a person’s life, it does not sever the relationship between the deceased and the living (Klass et al., 1996; Klass & Steffen, 2018). A continuing bond to the deceased presents freedom for the bereaved to continue to live meaningful lives without needing to relinquish what was lost (Klass et al., 1996), and the encouragement to connect to the deceased is often therapeutic rather than harmful (Neimeyer et al., 2014). While bereavement and loss are choiceless life events (Attig, 2011), it remains evident that death practices can support the bereaved in maintaining bonds to the deceased, and offer the potential opportunity for the bereaved to express their love in a way that is personally significant and meaningful (Kastenbaum, 2004; Neimeyer et al., 2014).

Death practices incorporate funerals, which are one of the oldest rituals known to humankind (Kastenbaum, 2004). Funerals and funeral services are an event where individuals, family, friends, and community members assemble to perform a death practice, such as burial or cremation, that memorializes the person who has died (Dennis, 2014). A primary task of a funeral is to provide social, psychological, physical, and spiritual support to the recently bereaved, while also honoring the deceased person’s life (Dennis, 2014). Funerals can offer the recently deceased a spiritual, cultural, and/or socially sanctioned rite of passage, and this may provide the bereaved with a sense of equanimity knowing they have fulfilled their social role (Kastenbaum, 2004). Yet, according to Neimeyer et al. (2014), “the story of the death itself and our changed relationship to the deceased are personally narrated, socially shared, and expressed in compliance with or contradiction to widely varying communal rules” (p. 486). As such, a person’s expression of grief may not necessarily align with particular socio-cultural norms that guide the funeral process in a given family context, which can result in considerable emotional turmoil for the bereaved (Hothschild, 2012). The lack of social recognition, validation, and support that a bereaved person may experience for violating socio-cultural expectations or rules has been conceptualized as ‘disenfranchised grief’, which excludes a bereaved person from public mourning and social support (Doka, 1989, 2002, 2020). As such, while death practices can often provide therapeutic opportunities for the bereaved, it is plausible to conceive how death practices can also be a place of adversity. This is especially palpable when acknowledging how adverse events may arise during a death practice and/or funeral from: Pre-existing family conflicts, a bereaved person’s wishes being ignored, an issue arising within the funeral home, or when there are significant financial pressures around the funeral (Gamino et al., 2000). Beyond thinking about death as it relates to the bereavement of the individual, a number of scholars have pointed to the socio-cultural relevance of death and death practices.

Death practices share many commonalities but are also unique across cultures and are imbued with a range of socio-cultural assumptions. According to Kastenbaum (1973), a ‘death system’ is a “sociophysical network by which we mediate and express our relationship to mortality” (p. 310). Death systems refer to the processes by which societies and cultures establish knowledge, attitudes, beliefs, and practices towards death. These death-related processes are influenced by people, places, times, objects, and symbols; death systems fulfill certain functions such as disposing of the dead and allowing various communities to make sense of death (Corr, 2015; Kastenbaum, 1973). Interestingly, death-related practices evolved differently in various societies and cultures around the world, and as such there are no universal and/or singular perspectives about how individuals must engage with funeral customs. The distinctness of different socio-cultural perspectives is important to highlight given that systematically established death practices within Western society can be a source of marginalization, as outlined later in this paper. Fundamentally, a consideration of death systems illustrates how bereavement and funeral rituals are embedded within social, cultural, and historical structures that are brimming with symbolic meaning (Corr, 2015; Kastenbaum, 1973). As a result, it becomes evident that death practices are continually being shaped and molded by socio-cultural assumptions within a particular society.

The socio-cultural meanings, roles, and the structural hierarchies of dominance that shape bereavement, the grieving response, and death practices have traditionally been under-valued in thanatological research (see Harris & Bordere, 2016; Thompson & Cox, 2017). Thanatological research has typically favored psychological epistemologies, which focus on an individual’s experience. While this research has been instrumental for the field, it is equally important to acknowledge that psychological phenomena do not occur in a social vacuum (Thompson et al., 2016). Thompson et al. (2016) propose a simple yet useful analogy of cream in coffee as a means to understand the symbiotic nature between an individual’s intrapsychic experience and their fundamentally social existence. If we think of society as coffee, and the individual as cream, we can see how an individual is unified and integrated within society, and how the two are deeply interwoven, rather than existing as distinctive binaries (Thompson et al., 2016; Thompson, 2020). Recognizing the social influences surrounding death practices becomes increasingly complex when we take into account the many social stratifications and social divisions that can influence a person’s grief and funeral practices. Social positionalities such as socioeconomic class, gender, ethnicity, dis/ability, sexual orientation, religion/spirituality, nationality, education, familial histories, cultural traditions, and other factors can influence how people grieve and how death practices are enacted. Socioeconomic class in particular is a powerful determinant of one’s experiences and options around death practices in the contemporary Western death industry; what a grieving person wishes or needs to do can be precluded by one’s financial situation (Gamino et al., 2000), and this will be further explored in ensuing sections.

## Questioning the Western Death Industry

Historically in Western societies, funeral rituals and practices have occurred at home and/or in religious settings (Aries, 1980), rather than professional corporatized settings. According to the historian Aries (1980), death was once viewed as a natural part of life and encountered with equanimity in the West; however, this perception changed over time, and in the modern period death became denied, invisible, medicalized, and something to be feared (Aries, 1980). Aries (1980) demarcates this era as ‘forbidden death,’ which illustrates how death becomes reprehensible and isolated in Western societies and provides the broad framework for understanding how professional death-care workers emerged within contemporary society. Further, it has become evident that the role of economic capital has become prominent in influencing social relationships, including relationships to the deceased in funeral rituals (Sanders, 2012). This is poignantly stated by Lynch (1999), who is both a funeral director and a poet:

A funeral is not a great investment; it is a sad moment in a family's history. It is not a hedge against inflation; it is a rite of passage. It is not a retail event; it is an effort to make sense of our mortality. It has less to do with actuarial profits and more to do with actual losses. It is not an exercise in salesmanship; it is an exercise in humanity (para. 9).

Lynch’s words capture how funeral practices can potentially embody meaningful and therapeutic practices for people who are recently bereaved, and how these practices can simultaneously be targeted by the exploitative nature of an industrialized and capitalistic death system. This is of great concern given that the human (and older) population is significantly expanding in Western society, and thus the funeral home and funeral service industry will likely continue to flourish and expand. The funeral industry has become a lucrative business; Audrebrand et al. (2018) state that “North Americans now fuel a multibillion-dollar industry that has transformed the dead into highly profitable revenue streams” (p. 1328). A select few corporations, which Smith (2007) refers to as *Big Death*, have purchased large numbers of regional and family-run funeral businesses in the last forty years; these corporations maintain a deceptive appearance of a local business by continuing to operate under the original funeral home name in the communities where they were initially established (Smith, 2007). These mega-corporations own thousands of funeral homes across North America; as a result, these businesses have tremendous economic power, resulting in inflated prices for funeral services with little accountability or regulation (Smith, 2007). Additionally, the term ‘traditional’ is often utilized in the modern funeral industry to encourage consumers to spend more money on a funeral service, and to justify the increase of services provided by the funeral home. Yet, there is nothing ‘traditional’ about a profit-driven funeral industry that charges exorbitant fees to manage our deceased loved ones; in fact, funerals are one of the largest expenses a person will incur in a lifetime (Kopp & Kemp, 2007). In Ontario, Canada, for instance, journalists have reported that some community members have described aggressive funeral sales practices that target recently bereaved individuals; that pre-paying for funerals is ineffective in avoiding expenses due to hidden fees; and caskets and urns are consistently subject to excessive price markups from wholesale price lists, ranging from 150% to 400% per item (Cribb et al., 2017).

While funeral prices, services, and practices vary across Western societies, many bereaved individuals and families are forced to make quick decisions regarding costly services in unimaginably strenuous and emotionally overwhelming times. Beyond the difficulties of making an informed financial decision within a raw state of grief, many individuals have a lack of experience regarding funerals due to the isolated nature of death within contemporary Western societies (Aries, 1980). Many grieving persons utilize few sources of information to make informed choices for the funeral service, as people are struggling to adjust to the complex process of navigating an entirely unfamiliar industry (Kopp & Kemp, 2007). What feels like an already impossible situation can be magnified by inadequate bereavement-related workplace laws to support time away from employment. For instance, the Government of Canada outlines in the  Canada Labour Code (2019) how workers are entitled to paid bereavement leave, but only for three days, and only if the worker has been employed for three months (if not, they are entitled to the three days without pay). The maximum of three days bereavement leave is also not applied if it occurs on an employee’s vacation, and/or if the death occurs on scheduled time-off; for example, if the worker’s loved one dies on a Friday evening after work, and if the worker’s days off were Saturday and Sunday, the only approved day off would be the Monday (Canada Labour Code, 2019). Disturbingly, this bereavement leave only includes death of a biological family member, spouse, and/or spouse’s family (Canada Labour Code, 2019). Colleagues, neighbors, close friends (including online friends), ex-spouses, animals, or even the death of a lifelong therapist/doctor are not entitled for bereavement leave. This narrow definition of grief also fails to acknowledge other loss experiences, and reflects the value of production over human wellness that is often present in industrialized, capitalistic, and Westernized nations (Harris, 2010; Shelvock, 2018). In the professional experience of the authors, some individuals will even return to work the same day a funeral has occurred, and will not even utilize the bereavement leave offered by the Canadian government; many people do not have the luxury of taking an extended leave of absence, and remaining in a position of favor with their employer is crucial for their survival. Consumer-driven values such as these can cause people to suppress their grief, which can create unnecessary stress and potentially impede adaptive responses to grief (Harris, 2010).

In identifying various socioeconomic power asymmetries that structurally exist in the funeral industry, it is our hope to challenge the ongoing rationalization of capitalistic practices in this context. Our point here is not to condemn modern death-care professionals, but rather to consider how we can best support those who may be experiencing significant losses and the suffering associated with the grieving process. It is conceivable that death-care workers may find themselves in a paradoxical role, as ethical standards are often in opposition to the orientation toward financial gain. Sanders (2012) explains this by stating, “the fiduciary realm places a different set of demands on those in the industry – to be rational (disinterested with regard to non-market-related concerns), opportunistic, and instrumentalist” (p. 279). Capitalistic pressures can create a moral dilemma for modern-day funeral service providers, potentially complicating the central role of the death-care professional, which is to support others in profoundly difficult situations that relate to death and grief. The danger of exploitation of grieving persons in such situations raises important questions about the industrialization of death-care and whether death practices might more productively be viewed within the realm of the continuum of health-care. Instead of utilizing a structural system that financially rewards the exploitation of recently bereaved persons who are in psychological and social distress, it may be advantageous for bereaved persons to have access to death-care services that are part of the social contract, and paid for through publicly-funded taxation. In Ontario, for instance, taxes are not collected on certain essential services such as medical care, dental care, and prescription medications. Yet, it is important to note that funeral services are taxed within Ontario, which implies that funeral services and/or body disposal is conceived as a non-essential service. However, the COVID-19 pandemic has confirmed how funeral and bereavement services are essential services for maintaining public wellbeing, as the world has experienced contagious viral infections and subsequently higher mortality rates (Van Overmeire & Bilsen, 2020). This further highlights the ambiguous nature of what point does health care end and death care begin, especially in relation to public trust and public funding. While the implications of constructing a new death-care system are beyond the scope of this paper, it appears advantageous to consider how removing the financial pressure on recently bereaved persons could be advantageous for supporting our collective health as a society, offering more legitimization towards the experience of bereavement and grief, and possibly for supporting more environmentally friendly funeral practices, as will be discussed.

## Consumption, Environmental Degradation, and Public Health

As outlined previously, the oppression from industrial capitalistic values in death practices can be detrimental to our individual and collective well-being; yet there is another disturbing phenomenon that is slowly occurring. In recent years, it has become increasingly clear how human activities are causing unprecedented and irreversible damage to the environmental systems we depend on. Humanity has altered the planet indefinitely, and capitalism is the propulsive force that is bolstering the ecological crisis; yet, this insight can create a form of paralysis when the urgent social, political, cultural, and existential implications are realized, as captured by evocative documentaries such as *Anthropocene: The Human Epoch* (Burtynsky et al., 2018). The established system of capital, which is constructed on infinite growth, is entirely unsustainable on a planet with finite resources, and subsequently results in extensive loss around the world (see Cunsolo & Landman, 2017; Nixon, 2011; Wallace-Wells, 2019). The reaction to this loss can be conceptualized as environmental and/or ecological grief, which is a natural response to the physical loss of ecosystems or animal species, identity loss from changing ecosystems that were once familiar, and the displacement of traditional environmental knowledge (Cunsolo & Ellis, 2018; Kevorkian, 2020). This grief can also be anticipatory in nature regarding forthcoming ecological losses. For example, in northern Canada, an Inuit community member captures this type of grief poignantly by stating, “Inuit are people of the sea ice. If there is no more sea ice, how can we be people of the sea ice?” (Cunsolo & Ellis, 2018, p. 277). The urgency for the development of new ways of thinking about death practices is underlined by this experience of ecological grief, and by the ongoing climate breakdown which has far-reaching and substantial consequences for all of life on Earth.

Warnings from ecologists and other biological scientists effectively illustrate how a sixth mass extinction is occurring (Barnosky, 2014; Ceballos et al., 2017). This is echoed by The Intergovernmental Panel on Climate Change (2014), a scientific organization under the auspices of the United Nations, who released a synthesis report highlighting the essential features of climate change that mirrors that of extinction: rising global temperatures; arctic ice, glaciers, and snow covers are melting; the warming and acidification of oceans, with sea levels rising; extreme weather patterns and events are becoming more frequent and destructive; irreversible changes are occurring to ecosystems, resulting in species extinction, destruction of agriculture, all of which have an impact on human health. The effects of climate change are unevenly distributed among humanity, as vulnerable individuals, disadvantaged communities, and developing countries with low incomes are at a far greater risk of potential burdens/harms (Climate Change Synthesis Report Summary for Policymakers, 2014). This is further supported by the National Aeronautics and Space Administration (NASA), as NASA reports that nearly two hundred scientific organizations worldwide have issued public statements endorsing this position, and scientific consensus states that the current ecological crisis is a direct result of human activities (NASA, 2020). To date, there has been a lack of research on how contemporary Westernized death practices may be contributing to the ongoing ecological crisis, a gap that we argue is of urgent concern, and that needs to be addressed.

While, as discussed earlier, there is no universal understanding of traditional death practices, the ‘traditional’ categorization of a funeral in Western society is often associated with a full-service funeral that involves a luxurious casket that is buried in the ground. Beyond the thousands of dollars caskets generally cost, they also pose a range of environmental and public health concerns. For instance, many caskets pose a risk as the metals utilized in their construction can deteriorate and corrode into harmful toxins (Olivier & Jonker, 2012). When buried in the earth, various contaminants that leach into the soil from wood caskets are varnishes, sealers, and wood preservatives; metal casket contaminants include steel, copper, zinc, and lead, which are poisonous to living creatures (Spongberg & Becks, 2000). The contamination of the soil is especially concerning where land and water can be polluted by the leachate from these caskets and thus have direct implications for human or animal health (Canning & Szmigin, 2010). This environmental hazard occurs in conjunction with the abundant amount of resources in the form of metal, wood, cloth, and cushioning that are required for the construction of an aesthetically appealing casket. Furthermore, caskets are often placed in a thick concrete burial vault in the ground in addition to a headstone above the grave, which highlights how these death processes utilize a tremendous amount of raw materials. These death practices not only alter the land in which burials occur, but there are also environmental consequences surrounding the maintenance of the cemetery. Urban cemeteries are often a monoculture of perfectly cut grass, which eliminates any biodiversity that may have previously existed. This is in addition to excessive land use and capacity restrictions, as burial plots are typically a one-time-use in North America (Canning & Szmigin, 2010).

Another concerning aspect of the ‘traditional’ burial is that the deceased are often embalmed. The majority of funeral directors across North America advocate for embalming corpses; however, others regard it as an unnecessary expense for a funeral service that can result in harm to human and environmental health (Doughty, 2014). Contemporary embalming practice in North America “replaces organic blood with various toxic and carcinogenic chemicals, particularly formaldehyde” (Chiappelli & Chiappelli, 2008, p. 24). Typically, embalming is conducted by creating an incision on a deceased person’s artery, draining the person’s blood which goes into the sewage system, and pumping embalming fluids into the person’s body (Chiappelli & Chiappelli, 2008; Doughty, 2014). The International Agency for Research on Cancer (2018) has found an excess number of deaths from nasopharyngeal cancer and leukemia for embalmers, funeral directors, pathologists, and anatomists who are exposed to formaldehyde. As a result, it is in the best interests of death-care workers’ health to not be exposed to the embalming process. The dangerous effects of embalming also extend to the environment, as an additional component of embalming fluid is phenol (Kleywegt et al., 2019). According to the Canadian Environmental Protection Act (2019), both phenol and formaldehyde are classified as highly toxic substances that are harmful to the environment. Chiappelli and Chiappelli (2008) also found that formaldehyde through waterborne exposure can damage and/or kill marine plant life, and that embalmed bodies at sea require an extended period of time to decompose because aquatic creatures and fish avoid these chemically infused bodies. Moreover, Kleywegt et al. (2019) found that aquatic environments experience higher concentrations of several contaminants near funeral homes that participate in embalming practices. Additionally, these environmental scientists have reported that embalming fluids can introduce illegal substances which are banned in some jurisdictions, such as Ontario, to the local aquatic environment (Kleywegt et al., 2019); this occurs because embalming fluid consists of a myriad number of preservatives and pesticides for delaying decomposition. It is also concerning to think of the risk of embalming fluids leaking from cemeteries into groundwater, as it unknown how long formaldehyde endures in the soil and/or how much contamination formaldehyde causes in the interim (Chiappelli & Chiappelli, 2008). If we are hoping to avoid ‘drinking’ our deceased loved ones, this might be enough reason to discontinue the embalming process (Chiappelli & Chiappelli, 2008).

While cremation has become a more common practice in North America due at least in part to lower financial fees and flexible options it offers grieving persons (Dennis, 2014), cremation can also be environmentally damaging. Cremation utilizes a significant amount of fuel consumption to sustain the high temperatures of the incinerator machinery; further, the cremation process releases a variety of chemical compounds and carbon emissions into the atmosphere (Canning & Szmigin, 2010). To illustrate this, consider how cremating embalmed bodies can be a way to directly release formaldehyde into the atmosphere, and how airborne exposure to formaldehyde can poison our bodies (Chiappelli & Chiappelli, 2008). Additionally, it is a common practice for people to be cremated with personal belongings as long as they are not explosive (Doughty, 2014). This can result in the release of additional toxic fumes from metals, plastic, and/or other pollutants into the air. Furthermore, dental amalgam can contain a significant amount of mercury, and cremations have been linked to the atmospheric pollution of mercury (World Health Organization [WHO], 2007, 2017). Mercury is an extremely toxic chemical and is considered one of the top hazardous chemicals to public and environmental health, as mercury affects the nervous, digestive, and immune system, in addition to having effects on lungs, kidneys, skin, and eyes (WHO, 2017). Mercury pollution also bioaccumulates in living organisms such as fish, which results in the direct consumption of mercury through our food sources (Sumner et al., 2019). In short, there are significant and undeniable environmental and public health concerns with cremation, embalming, and ‘traditional’ forms of burial in Western societies. Yet, conversations about the environmental implications of death practices appear to be taboo, marginalized, and largely silenced; many of the death practices in Western society relate to death denial and a lack of meaningful conversations about death being a natural part of life (Aries, 1980). This also reflects an outgrowth of capitalistic values, as capitalism has normalized a sense of stoicism which encourages the active repression of death, so that people may remain focused upon acquisition and production (Harris, 2010). However, a small pocket of emerging activists and scholars are working to bring these issues into the public forum in recent years.

## Returning to the Earth: Green Death Practices

Conversations about green, natural, and/or ecological burials and funerals are slowly becoming more prevalent, and environmental politics have begun to be interwoven with thanatological and end-of-life concerns. The social movement towards green death practices and the deep desire for sustainability is a direct response to the climate breakdown and ecological crisis currently altering the planet. As a result of this crisis, alternative ways to memorialize the deceased in which environmental and public health is prioritized have become more prominent concerns within contemporary society. The Green Burial Society of Canada (2020), which is a prominent non-governmental organization that advocates for, and promotes ecologically friendly death practices, defines green burials as:

A statement of personal values for those who seek to minimize their impact on the local and global environment. For people who are mindful of the cyclical nature of life, green burial is a spiritually fulfilling alternative to conventional burial or cremation. It is an environmentally sensitive practice: the body is returned to the earth to decompose naturally and contribute to new life. (para. 1)

As this quote illustrates, one objective of green burials is to minimize further damage, contamination, and pollution of the environment; additionally, many individuals perceive these death practices as a more intimate way to connect to the natural environment (Canning & Szmigin, 2010). Underlying this movement is the opportunity to align environmental and humanistic values with choices about practices that will surround one’s death. In this way, it may be plausible to contribute to the well-being and health of others and the planet. While environmentally sustainable death practices seem to vary considerably from region to region, such practices may include utilizing biodegradable coffins that are made from cardboard instead of hardwood, or utilizing environmentally friendly markers for memorializing purposes rather than granite or concrete tombstones (Brennan, 2014). Green or natural burials such as these could substantially reduce costs associated with burial, as there is no need for expensive caskets, embalming, or concrete vaults (Coutts et al., 2018). Green burial practices are guided by ecologically conscious principles, which suggest: i) deceased persons should not be embalmed; ii) deceased persons should receive a direct earth burial; iii) the burial should promote ecological restoration and conservation (i.e. helping grow the local eco-system); iv) communal memorialization is to be encouraged rather than individual memorialization, and memorization should use only natural materials; v) green burial cemeteries will optimize land use to avoid wasting valuable land (Green Burial Society of Canada, 2020). Woodland environments also appear to be advantageous for green cemetery spaces as opposed to placing green cemeteries in urban spaces. The use of woodland environments avoids further environmental pollution by eliminating the need to use pesticides, fertilizers, and/or fuel for machinery which is used to maintain urban cemeteries (Brennan, 2014). Green and natural cemeteries use far fewer resources than a traditional cemetery and are increasingly being utilized for land conservation efforts as a means to promote habitat restoration (Coutts et al., 2018). Cremation is often avoided in green death practices due to the negative impacts on environmental health as previously stated; however, this appears to be changing as alkaline hydrolysis, also known as water or flameless cremation, is becoming legalized across North America (Brennan, 2014). According to the Cremation Association of North America (2019), alkaline hydrolysis

uses water, alkaline chemicals, heat, and sometimes pressure and agitation, to accelerate natural decomposition, leaving bone fragments and a neutral liquid called effluent. The decomposition that occurs in alkaline hydrolysis is the same as that which occurs during burial, just sped up dramatically by the chemicals. The effluent is sterile, and contains salts, sugars, amino acids and peptides. (para. 3)

As a result, alkaline hydrolysis avoids releasing chemical compounds and carbon emissions into the air, which lowers greenhouse gases (Cremation Association of North America, 2019). Additionally, this process uses far less energy than fire-based cremation and allows for separation of dental amalgam for safe disposal to avoid mercury pollution (Brennan, 2014). Despite the slow legalization process pertaining to alkaline hydrolysis, the process has been tested for years by universities and hospitals to ensure the remaining contents are non-toxic; interestingly, pet crematories have used this process widely as they operate under different legislation (Cremation Association of North America, 2019). Green death practices may also consist of other practices, such as sea burials, tree burials (Canning & Szmigin, 2010), or sky burials where bodies naturally decompose via ecological processes (Doughty, 2014). Other emerging trends consist of mushroom burial suits, biodegradable tree pods, natural burials in conservation grounds, and composting human bodies in specialized facilities, among others. Unfortunately, there is a lack of formalized research and scholarship regarding these green death practices at this time. Nonetheless the considerations highlighted above show how green burials and ecologically friendly death practices offer hope for sustaining environmental and human health into the future.

Green death practices also come with their own challenges. Attempting to balance environmental sustainability while supporting equity and diversity synchronously can be challenging, as these are complex ethical issues. For example, it becomes increasingly evident that all death processes have social and political components to them, and that transforming the current death industry to only include environmentally sustainable practices may also create unintended social justice issues. It is clearly oppressive to deny individuals and communities the opportunity to grieve and memorialize their deceased loved ones in a way that is congruent with their values and needs; thus, green death practices may not universally align with every person.

Another potential limitation to in-land green burials (but certainly not all green death practices) is that green burials can encounter the same issue as other types of burial, which is land-capacity constraints (Canning & Szmigin, 2010). The recycling of graves is important for sustainability purposes. Yet, most legislation in North America does not support the re-use of graves, despite the limited duration of time for one grave before it is re-used being a common death practice throughout the world (Green Burial Society of Canada, 2020). Current legislation and laws need to be altered to support environmental and communal health by legalizing the recycling of graves.

Fully green cemeteries also remain highly inaccessible, as there are only a handful of green cemeteries that currently exist throughout North America. Travelling long distances to a fully green cemetery presents logistical and financial challenges in storing and transporting a deceased person’s body that does not undergo embalming. Green, natural, and ecologically friendly funerals are also vulnerable to becoming the next exercise in funeral salesmanship if structural changes aren’t implemented. Green death practices, which are typically rather simple and straightforward processes, are at risk of becoming increasingly more extravagant. This is a legitimate concern given evidence of how the contemporary funeral industry has evolved into a multi-billion-dollar industry, and how memorialization and leaving a legacy has become contorted by consumer culture (Coutts et al., 2018; Mitford, 1963). Green death practices may be fetishized and/or perceived as exotic because they are outside the ‘norm,’ and thus the demand of a new consumer trend may offer the opportunity for the death industry to create a new revenue stream. Since green funerals are currently inaccessible by the vast majority of the public, it is concerning to think how green burials could evolve into an exclusive option for only the rich and powerful. The current inaccessibility of green cemeteries could become homogenized as the next capitalistic practice in the death industry, and exorbitant fees could be charged since only some cemeteries offer this service. If this is to be prevented, our society needs to create and invest in more sustainable death practices as a whole, and to continually work towards removing death-care from privatized corporatized care. While death practices can operate as symbols of power, these symbols can be reclaimed to ensure that green death practices remain available, accessible, flexible, and accommodating to recently bereaved persons.

## Conclusion

This paper has focused on explicitly outlining the ongoing issues of economically driven and environmentally destructive death systems and practices, with the goal of attempting to lay a foundation for a new social imagination around dying and death in Western societies. Current funeral and death practices rooted in capitalistic values tend to obscure the social importance of death, resulting in death as an opportunity for profit at the expense of bereaved families and the environment. Transitioning funeral services from the privatized and corporate sector to a publicly funded sector could ensure better equity in access to services, in addition to offering more protection to potentially marginalized bereaved individuals and to the environment.

New ways of conducting death practices and existing on this planet are urgently required, as without such action, the planet is in peril (see Nixon, 2011; Wallace-Wells, 2019). In demonstrating how contemporary death practices and rituals are socially, historically, and culturally situated practices that are constantly evolving and fluid in nature, it is our hope to emphasize how death systems can be re-imagined (Corr, 2015; Kastenbaum, 1973). Change is possible, but it is also clear that replication of every-day death practices without critically reflecting on the status quo will ensure things never change. Public education about green death practices can serve as a starting point for a much larger and longer social project surrounding the implementation of structural, political, and social change. It is plausible to suggest that engaging in critical reflection on the funeral industry and the environmental implications of death practices in Western societies has the potential to inform positive change at a societal level. Professional education researchers outline how developing critical reflection can assist individuals and social groups to align actions with values (Kinsella & Pitman, 2012). By refusing to uncritically accept harmful traditions, a plethora of opportunities emerge for which critical reflection can be utilized to alter the unique contexts in which we find ourselves, and this could promote well-being and social justice (Kinsella, 2012). Opening conversations about what green death practices entail, and how they may offer pathways to honor our relationships to the planet, other human beings, and even our own deepest values, offers a potentially fruitful way forward.
